# A Multi-Targeted Strategy for Relapsed/Refractory B-Cell Non-Hodgkin Lymphoma: Real-World Outcomes of the ViPOR Regimen

**DOI:** 10.3390/jcm15145401

**Published:** 2026-07-10

**Authors:** Süreyya Yiğit Kaya, Mehrad Vatani, Senem Maral, Hüseyin Saffet Beköz, Amir Hossein Abedi, Olgu Erkin Çınar, Beyza Oluk, Leylagül Kaynar

**Affiliations:** 1 Department of Hematology, Medipol Mega University Hospital, Istanbul Medipol University, 34214 Istanbul, Turkey; senemmaral@gmail.com (S.M.); hsynbkz@yahoo.com (H.S.B.); olgu.cinar@medipol.edu.tr (O.E.Ç.);; 2 Faculty of Medicine, Istanbul Medipol University, 34810 Istanbul, Turkey; mehradvt@gmail.com; 3 Department of Internal Medicine, Medipol Mega University Hospital, Istanbul Medipol University, 34214 Istanbul, Turkey; abedimd@outlook.com; 4 Department of Hematology, Kocaeli City Hospital, 41060 Kocaeli, Turkey; senbeyza@gmail.com

**Keywords:** ViPOR, allogeneic stem cell transplantation, diffuse large b-cell lymphoma, follicular lymphoma, relapsed/refractory, B-cell non-Hodgkin lymphoma

## Abstract

**Background/Objectives**: Relapsed/refractory (R/R) B-cell non-Hodgkin lymphomas (B-NHLs) remain a major therapeutic challenge, particularly in patients with aggressive subtypes and limited treatment options after multiple lines of therapy. The ViPOR regimen, a multi-targeted combination of venetoclax, ibrutinib, prednisone, obinutuzumab, and lenalidomide, has demonstrated promising activity in early-phase studies; however, real-world data are limited. **Methods**: We conducted a retrospective, two-center study including patients with R/R B-NHL treated with the ViPOR regimen between January 2024 and April 2026. Clinical characteristics, treatment responses, survival outcomes, and safety data were analyzed. **Results**: A total of 14 patients were included, with a median age of 45 years and a median of 3.5 prior lines of therapy. Most patients had advanced-stage disease (71% stage IV), and 36% had primary refractory disease. The interim overall response rate (ORR) was 62%, including 31% complete response (CR) and 31% partial response. At the end of treatment, the ORR was 54%, with a CR rate of 54%. Radiotherapy was incorporated in selected patients with residual disease and contributed to response deepening. Six patients (43%) were successfully bridged to allogeneic stem cell transplantation, all in CR. Responses were notably more favorable in the activated B-cell (ABC) subtype compared to the germinal center subtype. The most common adverse events were neutropenia (57%) and diarrhea (36%), and the regimen demonstrated a manageable safety profile. During follow-up, 57% of patients remained alive. **Conclusions**: The ViPOR regimen demonstrated promising efficacy and acceptable safety in heavily pretreated R/R B-NHL, particularly in the ABC subtype. Its ability to induce deep responses and enable bridging to allogeneic stem cell transplantation highlights its potential role in real-world clinical practice. Larger prospective studies are warranted to confirm these findings.

## 1. Introduction

B-cell non-Hodgkin lymphomas (B-NHLs) are a heterogeneous group of malignancies that range from indolent to highly aggressive subtypes [1]. Across the spectrum of aggressive B-NHLs, particularly in advanced-stage treatments, outcomes following relapse or resistance remain poor, with an objective response rate of 26% and a median overall survival of 6.3 months being reported [2,3]. Although salvage chemotherapy followed by autologous stem cell transplantation (ASCT) remains an important option for some patients, many are ineligible or relapse thereafter. Meanwhile, chimeric antigen receptor T-cell (CAR-T) therapy has improved outcomes in relapsed/refractory (R/R) large B-cell lymphoma; however, access remains limited and outcomes after treatment failure are poor [4,5]. A major biological challenge encountered in aggressive B-NHLs is the simultaneous activation of multiple survival pathways, particularly B-cell receptor signaling and anti-apoptotic mechanisms such as BCL2, which may limit the efficacy of single-agent therapies and support the use of rational combination strategies [6,7].

The ViPOR regimen, which combines venetoclax, ibrutinib, prednisone, obinutuzumab, and lenalidomide, is designed to target complementary survival mechanisms simultaneously. Both in early-phase trials and in limited real-world data, durable responses have been observed in patients with R/R B-NHL; it is thought that the ViPOR regimen may be effective in patients who have received intensive prior treatment and may serve as a bridge to subsequent cell therapies [8,9,10,11]. However, real-world evidence remains limited. Therefore, we aimed to evaluate the efficacy and safety of ViPOR in patients with R/R B-NHL and to explore its role in real-world clinical practice, including its use as a bridging strategy to subsequent therapies, in a two-center cohort.

## 2. Materials and Methods

### 2.1. Study Design and Patient Selection

This retrospective study included patients with R/R B-NHL who were treated with the ViPOR regimen at two different centers. Eligible patients had a histologically confirmed diagnosis of B-NHL and received the ViPOR regimen between January 2024 and April 2026 at one of the participating centers. Patients with incomplete clinical data or missing follow-up information were excluded from the analysis. No patients were excluded on the basis of treatment response, disease course, or clinical outcome. Demographic and clinical data, including baseline disease characteristics, prior treatment history, extranodal involvement, and history of stem cell transplantation, were recorded using a standardized data collection form. Cell-of-origin classification (ABC versus GC subtype) was determined according to local pathology reports using an immunohistochemistry-based Hans algorithm [12].

### 2.2. Treatment Protocol

The ViPOR regimen (venetoclax, ibrutinib, prednisone, obinutuzumab, and lenalidomide) was administered following the principles of the regimen reported by Melani et al. [8], with minor modifications according to institutional practice. Treatment was delivered in 21-day cycles and continued for a maximum of six cycles or until disease progression, unacceptable toxicity, or transition to stem cell transplantation prior to completion of therapy. Supportive treatments, including antimicrobial prophylaxis and growth factor support, were administered according to institutional practice.

### 2.3. Response Evaluation and Safety Assessment

Radiologic assessment was performed in all patients using positron emission tomography–computed tomography (PET-CT). Treatment response was assessed according to the Lugano classification. Responses were categorized as complete response (CR), partial response (PR), stable disease (SD), or progressive disease (PD). Interim response assessment was performed after 2–3 cycles of treatment and the final response was evaluated at the end of therapy. Adverse events were graded according to the Common Terminology Criteria for Adverse Events (CTCAE), version 5.0. When concomitant medications with the potential for clinically relevant drug–drug interactions were required, doses of ViPOR components were adjusted according to international recommendations and institutional practice.

### 2.4. Ethical Considerations

This study was conducted in accordance with the Declaration of Helsinki and Good Clinical Practice guidelines. Ethical approval was obtained from Istanbul Medipol University Ethics Committee (date: 9 April 2026, number: 623).

### 2.5. Statistical Analysis

Descriptive statistics were used for all analyses. Continuous variables are reported as median (range), while categorical variables are expressed as number (n) and percentage (%). Overall survival (OS) was defined as the time from the date of first ViPOR administration to the date of death from any cause. Patients who remained alive at the time of data cutoff were censored at their last known date alive. Progression-free survival (PFS) was defined as the time from first ViPOR administration to the date of either documented disease progression (progressive disease, PD) or death from any cause, whichever occurred first. Patients without PD or death by the data cutoff were censored at the date of their last tumor assessment. In addition, if a patient initiated a subsequent anticancer therapy prior to experiencing PD or death, they were censored at the date of their final tumor evaluation. PFS and OS were estimated using the Kaplan–Meier method. Statistical analyses were performed using standard statistical software (SPSS Version 22). Artificial intelligence (AI)-assisted language editing tools were used solely for minor English language and grammatical editing during manuscript preparation.

## 3. Results

### 3.1. Patient Characteristics

A total of 14 patients treated with ViPOR across two centers were included. Baseline demographic and clinical characteristics are summarized in Table 1. The median age was 45 years (range, 22–76), and 50% of patients were female. Histological subtypes included diffuse large B-cell lymphoma, activated B-cell subtype (DLBCL, ABC; n = 8, 57%), diffuse large B-cell lymphoma, germinal center subtype (DLBCL, GC; n = 4, 29%), large B-cell lymphoma with IRF4 rearrangement (n = 1, 7%), and follicular lymphoma (FL; n = 1, 7%). Patient 3 had transformation from grade 2 FL to DLBCL, ABC, whereas Patient 2 had been initially diagnosed with classical Hodgkin lymphoma (cHL) before subsequently developing DLBCL, GC subtype.

At enrolment, nine patients (64%) had relapsed disease, whereas five (36%) had primary refractory disease. Among the five patients with primary refractory disease, CR was achieved in two (40%), SD in one (20%), and PD in two (40%). Of the nine patients with relapsed disease, one was not evaluable due to early death; among the remaining eight evaluable patients, CR was achieved in five (62.5%), SD in two (25%), and PD in one (12.5%). Baseline performance status was favorable, with 13 patients (93%) having an Eastern Cooperative Oncology Group (ECOG) score of 0–1. Most patients had advanced-stage disease, with Ann Arbor stage IV observed in 10 patients (71%). According to disease-specific prognostic indexes, the majority of patients had low- or intermediate-risk diseases. Comorbidities were limited, with 11 patients (79%) having no documented comorbid conditions.

### 3.2. Prior Treatment Characteristics

Prior treatment exposure and disease characteristics before ViPOR initiation are detailed in Table 1. Patients had received a median of 3.5 previous lines of therapy (range, 2–6). Extranodal involvement at the time of ViPOR initiation was documented in 10 patients (71%), including bone, lung, tongue, breast, skin, liver, gluteal muscle, adrenal gland, and kidney involvement. Bulky disease was present in four patients (29%), and the median lactate dehydrogenase (LDH) level was 287 U/L (range, 104–1211).

Three patients (21%) had previously received lenalidomide-based therapy, and two had prior exposure to both lenalidomide and obinutuzumab. Five patients (36%) had previously undergone ASCT. Four patients (29%) experienced relapse after ASCT despite achieving CR, with remission durations ranging from 6 to 22 months. One heavily pretreated patient (Patient 7) proceeded to allo-SCT after ASCT, as CR was first achieved only following transplantation and the risk of subsequent relapse was considered high.

### 3.3. ViPOR Regimen

ViPOR was administered in 21-day cycles. Methylprednisolone 100 mg was given intravenously or orally on days 1–7. Obinutuzumab 1000 mg was administered on days 1, 2, 8, and 15 of cycle 1, and on day 1 of subsequent cycles. Lenalidomide 15 mg and ibrutinib 560 mg were both given on days 1–14. Venetoclax was administered with a 12-day ramp-up schedule to a target dose of 400 mg. In patients without bulky disease, venetoclax was initiated in cycle 1, whereas in patients with bulky disease, it was initiated in cycle 2 after tumor reduction was achieved with the first cycle. Ramp-up dosing was performed in all patients. ViPOR was administered at a median of 4.5 lines (range, 3–7).

### 3.4. Treatment Response

The treatment responses and clinical outcomes are summarized in Table 2. Interim assessment to ViPOR could not be evaluated in one patient. This patient (Patient 2), who had previously been diagnosed with cHL and subsequently developed DLBCL, GC subtype, died during the first cycle due to disease progression. Among the 13 evaluable patients, CR was achieved in four (31%), PR in four (31%), SD in three (23%), and PD in two (15%), resulting in an interim overall response rate (ORR) of 62%.

At the end of ViPOR treatment (median, three cycles; range, 1–6), CR was achieved in seven of 13 evaluable patients (54%). Three patients (23%) with an interim PR subsequently deepened to CR, whereas one patient (Patient 8) progressed from PR to PD. SD and PD were each observed in three patients (23%). The ORR at the end of ViPOR treatment was 54%, with all responders achieving CR. To facilitate comparison between cell-of-origin subtypes, treatment responses and clinical outcomes according to ABC and GC classification are summarized in Table 3.

Radiotherapy (RT) was added into the regimen for three patients (21%) due to PR at interim assessment, all of whom had DLBCL of the ABC subtype. Patient 1 demonstrated PR at interim evaluation, with approximately 50% reduction in a bulky mediastinal mass. RT was initiated to the residual mass while ViPOR was continued concurrently, completing a total of five cycles. CR was achieved at the end of treatment, and the patient subsequently proceeded to allo-SCT. Patient 3 showed CR in most involved areas at interim assessment, but residual disease was present in the inguinal and iliac lymph nodes, consistent with a PR. RT was administered to these partially responding nodes and one additional cycle of ViPOR was given concurrently. CR was subsequently achieved and the patient underwent allo-SCT. Similarly, Patient 10 demonstrated CR in most involved areas with residual PR in the inguinal and iliac lymph nodes. RT was administered to the residual nodal disease. The patient subsequently achieved CR and proceeded to allo-SCT.

One patient with DLBCL, ABC subtype (Patient 6) achieved CR but could not proceed to allo-SCT because cerebral aspergillosis developed during the fourth cycle of ViPOR, leading to treatment discontinuation. At the end of ViPOR treatment, six of 14 patients (43%) were successfully bridged to allo-SCT, all of whom were in CR. After allo-SCT, Patient 1 (DLBCL, ABC) remained in CR at 13 months, Patient 3 (DLBCL, ABC) at 8 months, and Patient 4 (LBCL with IRF4 rearrangement) at 10 months. Patients 9 (FL) and 13 (DLBCL, ABC) are currently in the early post-transplant period and response assessment has not yet been performed. Patient 10 (DLBCL, ABC) died on day +31 post-allo-SCT due to sepsis before response evaluation could be performed.

### 3.5. Survival and Safety

Adverse events observed during ViPOR treatment are summarized in Table 4. The most common hematologic adverse events were neutropenia (57%) and thrombocytopenia (14%). Granulocyte colony-stimulating factor (G-CSF) was administered when clinically indicated. The most frequent non-hematologic adverse events were diarrhea (36%), hypokalemia (21%), and infections (14%). CNS aspergillosis occurred in one patient (Patient 6), resulting in treatment discontinuation during the fourth cycle. The patient developed hemiplegia, was treated with amphotericin B, and remained alive for 8 months without further lymphoma-directed therapy before discontinuing follow-up at their own request. In one patient (Patient 12), treatment was interrupted during the first cycle because of adverse events. Pulmonary thromboembolism (PTE) developed in one patient (Patient 7). No cases of tumor lysis syndrome (TLS) were observed.

All patients received valacyclovir/acyclovir and trimethoprim–sulfamethoxazole (TMP-SMX) prophylaxis. Two patients received entecavir prophylaxis because of anti-HBc positivity, and one patient received prophylactic enoxaparin. One patient had cryptococcal diarrhea at treatment initiation and received concomitant fluconazole; therefore, the doses of venetoclax (200 mg) and ibrutinib (280 mg) were reduced.

**Figure 1:**
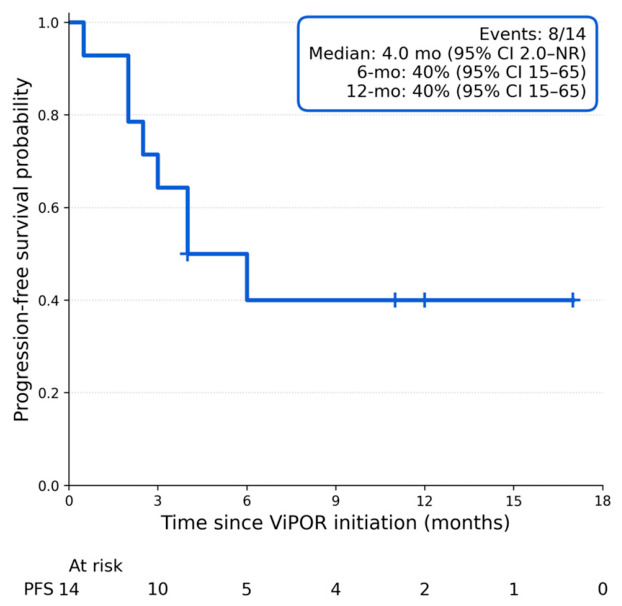
The median PFS was 4.0 months (95% CI, 2.0–NR).

**Figure 2:**
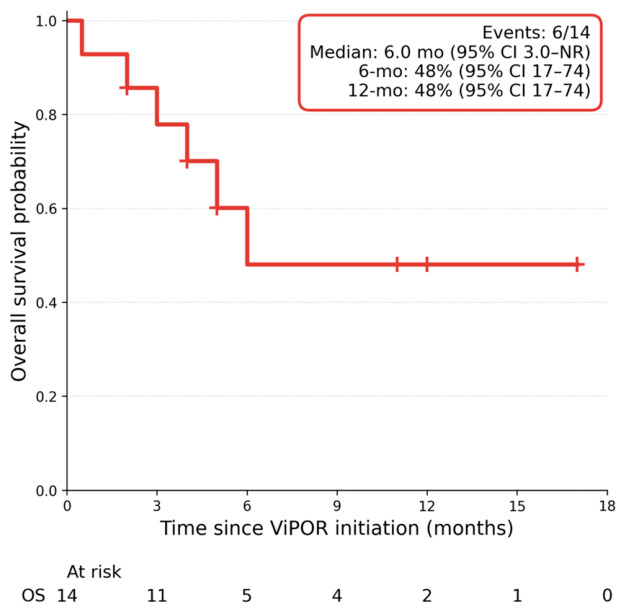
The median OS was 6.0 months (95% CI, 3.0–NR).

The median follow-up duration was 4.5 months (range, 0.5–17 months). The median PFS was 4.0 months (95% CI, 2.0–NR) (Figure 1) and the median OS was 6.0 months (95% CI, 3.0–NR) (Figure 2). The estimated PFS rates at 6 and 12 months were 40% (95% CI, 15–65%). The estimated OS rates at 6 and 12 months were 48% (95% CI, 17–74%). During follow-up, 6 of 14 patients (43%) died, of whom four died due to lymphoma progression; three had DLBCL, GC subtype and one had DLBCL, ABC subtype. One patient died due to PTE at the end of the second cycle (Patient 7, DLBCL, GC). Another patient died on day +31 post-allo-SCT due to sepsis (Patient 10, DLBCL, ABC). Overall, 8 of 14 patients (57%) remained alive at the time of analysis.

## 4. Discussion

The ViPOR regimen combines agents that target multiple complementary pathways involved in lymphoma cell survival. Ibrutinib disrupts B-cell receptor (BCR)-dependent signal transduction, which plays a central role in the survival of malignant B cells by inhibiting Bruton’s tyrosine kinase (BTK) [13,14,15]. Venetoclax selectively inhibits the anti-apoptotic protein BCL2, thereby eliminating the mechanism of apoptosis resistance frequently observed in B-cell lymphomas [16,17]. Lenalidomide exerts immunomodulatory and transcriptional effects through the cereblon-mediated degradation of transcription factors such as IRF4 and SPIB, leading to the suppression of NF-κB signaling and impairment of lymphoma cell survival [18,19]. Additionally, it has been shown that glucocorticoids such as prednisone inhibit oncogenic BCR signaling by modulating receptor expression and downstream signaling components [20]. Obinutuzumab, a type II anti-CD20 monoclonal antibody, can further enhance anti-tumor activity by promoting direct cell death and modulating BCR-associated signaling pathways [21]. The combination of these agents enables the simultaneous targeting of survival mechanisms and results in synergistic anti-tumor effects. Preclinical studies have shown that the simultaneous inhibition of BTK and BCL2 increases cytotoxicity compared to single-agent approaches and may help to overcome resistance mechanisms [16]. Similarly, it has been shown that lenalidomide enhances the activity of BCR pathway inhibitors by additionally suppressing NF-κB signaling and IRF4 expression [18]. Furthermore, BCL2 inhibition may enhance the efficacy of anti-CD20-targeted therapies and contribute to the elimination of lymphoma cells in experimental models [17].

The ViPOR regimen demonstrated consistent anti-lymphoma activity in heavily pretreated R/R DLBCL populations. Melani et al. first described the ViPOR regimen in a Phase 1b study, presenting a multi-targeted combination strategy for R/R B-NHL and laying the foundation for subsequent clinical development studies [22,23,24,25,26]. In the later Phase 1b/2 study by Melani et al., heavily pretreated patients (median three prior lines) achieved an ORR of 54% and CR rate of 38%, with 2-year PFS and OS of 34% and 36%, respectively, and a manageable toxicity profile [8]. In parallel, Melani et al. also reported the high efficacy of the ViPOR regimen in R/R FL, with an ORR of 100% and a CR rate of 79%, including high-risk patients such as POD24. MRD negativity was frequent and associated with improved PFS. The 2-year PFS and OS rates were approximately 51% and 73%, respectively [24]. The subsequent ViPOR-P trial (n = 40) incorporating polatuzumab increased efficacy to ORR 78% and CR 56%, with median 2-year PFS of 41% and OS of 53% after 28 months of follow up [27]. Real-world analyses included older and more heavily pretreated populations: a 2024 multicenter cohort (n = 48) had a median age of 61, median of four prior therapies, and 79% refractory disease; among patients completing ≥2 cycles, the median PFS was 9 months and OS 12 months [10]. A multicenter study (n = 56) of a highly frail ITT cohort reported a median PFS of 4 months and OS of 5 months, with a 12-month PFS of 43% and OS of 25%; outcomes improved in patients receiving ≥2 cycles, reaching a 12-month PFS of 55% and OS of 42% [9].

In our cohort (n = 14), patients were younger (median age 45), had a median of 3.5 prior treatment lines, and the disease was predominantly at an advanced stage (71% stage IV). Compared with previously published real-world cohorts, our population was younger, which may have contributed to better treatment tolerance, higher transplantation eligibility, and more favorable outcomes. A quarter of the patients had been exposed to lenalidomide, and in these patients, CR was achieved with ViPOR treatment, enabling allo-SCT. In the interim response assessment conducted after 2–3 cycles, the ORR was found to be 62% (CR 31%, PR 31%), and at the end of treatment, the ORR was 54% (CR 54%). RT was administered as consolidative treatment to three patients (21%) with residual disease identified at interim assessment while ViPOR therapy was continued. All three patients subsequently achieved CR by the end of treatment. Given the concurrent administration of additional ViPOR cycles and RT, the specific contribution of RT to response deepening cannot be determined. Nevertheless, RT was temporally associated with conversion from partial to complete response in these heavily pretreated, high-risk patients, particularly in those proceeding to allo-SCT. Six of 14 patients (43%) were successfully bridged to allo-SCT and five remained alive at follow-up. The treatment was generally well tolerated; the most common adverse events were neutropenia (57%) and diarrhea (36%). Overall, 43% of patients died; the primary cause of death was lymphoma progression. In our cohort, the median PFS was 4.0 months, and median OS was 6.0 months. This likely reflects the relatively short follow-up duration in our study, as well as early deaths among GC-subtype patients. Survival follow-up remains limited in some patients, but early complete remissions following transplantation are highly encouraging for this patient group.

CR was achieved in both the patient with FL and the patient with large B-cell lymphoma with IRF4 rearrangement. The patient with FL represented a high-risk case and had POD24 disease. The remaining patients had DLBCL. Among patients with DLBCL, the treatment responses differed markedly between the ABC and GC subtypes. Patients with DLBCL, ABC subtype generally demonstrated favorable responses (five CR, one PD, and two SD). In contrast, the GC subtype was associated with lower response rates and a higher risk of progression; all GC subtype cases exhibited stable or progressive disease, and all four patients with GC subtype died during follow-up. Notably, the majority of deaths due to lymphoma progression occurred in patients with GC subtype disease. Although the small cohort size is a limitation of our study, the response pattern observed in this series appears consistent with the subtype-specific activity of the ViPOR regimen previously reported to support GC/ABC biology [8,9,10,27].

Real-world analyses further support the subtype-dependent activity of the ViPOR regimen. Patients with DLBCL of the ABC subtype demonstrated substantially higher responsiveness than those with the GC subtype, with ORRs reaching 71% in the intention-to-treat cohort. No responses were observed in GC patients [9]. Successful bridging to CAR-T or allo-SCT was predominantly achieved in ABC patients; in a dedicated bridging-to-CAR-T subcohort, patients with DLBCL, ABC subtype attained a 100% ORR [9]. Sustained therapy (≥2 cycles) improved outcomes in DLBCL, ABC subtype, with a 12-month PFS of 55% reported in the multicenter study [9]. Similarly, a 2024 retrospective real-world analysis indicated that bridging and sustained responses were primarily observed in patients enriched for ABC/non-GC biology [10].

This subtype-specific activity is supported by the known biology of individual ViPOR components. Ibrutinib shows preferential activity in DLBCL, ABC subtype with an ORR of 37–40% compared to minimal responses in DLBCL, GC in multicenter Phase 1/2 and Phase 2 trials [13,14]. Preclinical evidence further indicates that DLBCL, ABC lines are more sensitive to BTK inhibition than GC lines [15]. Similarly, lenalidomide exhibits greater efficacy in DLBCL, ABC by downregulating IRF4 and SPIB, disrupting BCR-dependent NF-κB signaling, and inducing a toxic interferon response, while having minimal effects in DLBCL, GC models [18,20]. These findings provide a mechanistic rationale for the observed subtype-specific responses to ViPOR, highlighting the importance of targeting pathways critical for DLBCL, ABC.

The ViPOR regimen demonstrated both direct anti-lymphoma activity and utility as a bridging strategy to CAR-T or allo-SCT [8,9,10,27]. In our cohort, six of 14 patients (43%) were successfully bridged to allo-SCT following ViPOR-based therapy, consistent with these prior observations and supporting the utility of ViPOR as a bridging strategy to consolidative cellular therapies in R/R B-NHL.

In one patient (Patient 7), PTE developed during treatment. The patient had a prior history of deep vein thrombosis secondary to lymph node compression and had completed an appropriate course of therapeutic-dose anticoagulation at that time. No inherited thrombophilia was identified. Given this history, prophylactic low-molecular-weight heparin was administered throughout ViPOR treatment; however, PTE recurred despite prophylaxis. The ViPOR therapy was not discontinued because of the thromboembolic event. Only one patient discontinued treatment due to a severe infectious complication (CNS aspergillosis), while no other patients permanently discontinued therapy because of treatment-related toxicity. Hematologic toxicities, particularly neutropenia, were the most common adverse events, in line with prior phase 1b/2 and real-world analyses [8,9,10,22,23,24]. Infectious complications occurred in a minority of patients, including one case of CNS aspergillosis. Real-world data have reported rare but severe infectious complications, including fatal cerebral aspergillosis, which is consistent with our observation and highlights the risk of opportunistic infections in heavily pretreated patients [10]. Similarly, invasive CNS aspergillosis has been reported in a heavily pretreated patient receiving ViPOR-P, leading to treatment discontinuation despite antifungal therapy and subsequent death due to disease progression [10]. Invasive aspergillosis is a recognized but uncommon complication of ibrutinib-based regimens, and these findings support the consideration of antifungal prophylaxis in high-risk patients. Intravenous immunoglobulin replacement was administered in two patients (Patients 9 and 12) due to hypogammaglobulinemia and recurrent infections. Notably, immunoglobulin monitoring and IVIG use have not been systematically evaluated in published ViPOR studies, despite the use of multiple B-cell-directed agents, highlighting an important gap in the current literature. Based on our experience, the successful administration of ViPOR requires appropriate infection prophylaxis, close monitoring for opportunistic infections, and the careful management of drug–drug interactions, particularly with azole antifungals that may necessitate dose adjustments of venetoclax and ibrutinib. The ViPOR regimen demonstrated a manageable safety profile.

### Study Limitations

This study has certain limitations, such as a small sample size and a retrospective design. Furthermore, the inclusion of heterogeneous histological subtypes and disease characteristics in the study may have influenced treatment outcomes. The relatively short follow-up period also limits the ability to assess long-term survival outcomes. Another limitation is that radiotherapy was administered concurrently with ongoing ViPOR treatment in three patients with residual disease; consequently, its specific contribution to subsequent response deepening cannot be reliably distinguished from the effect of continued systemic therapy.

## 5. Conclusions

The ViPOR regimen demonstrated promising efficacy and acceptable safety in R/R B-NHL, particularly in the ABC subtype, and enabled bridging to allo-SCT in a substantial proportion of patients. Our findings also suggest that accurate cell-of-origin classification, especially in DLBCL, may be important for identifying patients most likely to benefit from ViPOR-based therapy. These findings support its role as a practical treatment option in real-world settings, although larger prospective studies are needed.

## Figures and Tables

**Table 1:** Baseline demographic and clinical characteristics.

Case	Age/Gender	Subtype	Stage (Ann Arbor)	Prognostic Index	Disease Status at Enrollment	ECOG/Comorbidity	Prior Therapies and Responses
1	31/M	DLBCL, ABC	2	IPI Low	Primary Refractory	0/None	6 × R-CHOP (SD) 2 × R-DHAP (SD) 2 × R-ICE (SD) 1 × R-GEMOX (PD)
2	28/M	DLBCL, GC (prior cHL)	2	IPI Low	Relapsed	1/None	6 × ABVD (CR) 2 × GDP (SD) 2 × MINE (SD) 2 × R-ICE (PR)
3	46/F	DLBCL, ABC (transformed from FL, FLIPI High)	4	IPI High	Primary Refractory	1/None	4 × R (SD) 2 × O + CHOP (PR) 2 × Bendamustine (SD) 4 × R-Bendamustine (CR) 1 × R-Lenalidomide (PD)
4	48/M	Large B-cell lymphoma with IRF4 rearrangement	2	IPI Low	Relapsed	1/None	6 × R-CHOP (CR) 2 × R-ICE (CR) ASCT (CR) 2 × R-GEMOX (PR)
5	44/F	DLBCL, GC	3	IPI Low-Intermediate	Primary Refractory	1/None	3 × R-CHOP (SD) 2 × R-GEMOX (SD)
6	59/F	D1LBCL, ABC	4	IPI Low-Intermediate	Relapsed	1/Hypothyroidism	6 × R-CHOP (CR) 2 × R-DHAP (PR) 2 × R-GEMOX (SD)
7	26/M	DLBCL, GC	4	IPI Low-Intermediate	Primary Refractory	1/Sleeve gastrectomy	6 × R-CHOP (PR) 4 × R-ICE (SD) 2 × R-GEMOX (PD) 1 × R-HYPERCVAD B + RT (PR) ASCT Allo-SCT (CR)
8	35/F	DLBCL, GC	4	IPI Low-Intermediate	Relapsed	1/None	6 × R-CHOP (CR) 2 × R-DHAP (PR) 2 × R-ICE (CR) ASCT (CR) 2 × R-GEMOX (SD)
9	39/M	FL	4	FLIPI Intermediate	Relapsed	1/None	6 × R-CHOP (CR) 5 × O-Lenalidomide (PR) 2 × R-ICE (SD)
10	60/F	DLBCL, ABC	4	IPI Low	Relapsed	1/None	6 × R-CHOP (CR) 2 × R-ICE (SD) 2 × R-Lenalidomide (CR) ASCT (CR)
11	76/F	DLBCL, ABC	4	IPI High-Intermediate	Relapsed	1/DM, HTN	6 × R-CHOP (CR) 2 × GEMOX (SD)
12	59/M	DLBCL, ABC	4	IPI High-Intermediate	Relapsed	1/None	6 × R-CHOP (CR) 2 × R-ICE (PR) 2 × R-GEMOX (PD)
13	22/F	DLBCL, ABC	4	IPI Low	Relapsed	1/None	6 × R-CHOP (CR) 2 × R-ICE (CR) ASCT (CR)
14	47/M	DLBCL, ABC	4	IPI High-Intermediate	Primary Refractory	3/None	6 × R-CHOP (SD) 2 × R-GEMOX (PR)

Abbreviations: DLBCL, diffuse large B-cell lymphoma; ABC, activated B-cell; GC, germinal center; FL, follicular lymphoma; FLIPI, Follicular Lymphoma International Prognostic Index; IPI, International Prognostic Index; ECOG, Eastern Cooperative Oncology Group; CR, complete response; PR, partial response; SD, stable disease; PD, progressive disease; R-CHOP, rituximab, cyclophosphamide, doxorubicin, vincristine, prednisone; R-DHAP, rituximab, dexamethasone, high-dose cytarabine, cisplatin; R-ICE, rituximab, ifosfamide, carboplatin, etoposide; R-GEMOX, rituximab, gemcitabine, oxaliplatin; ABVD, doxorubicin, bleomycin, vinblastine, dacarbazine; SCT, stem cell transplantation; ASCT, autologous stem cell transplantation; Allo-SCT, allogeneic stem cell transplantation; RT, radiotherapy; DM, diabetes mellitus; HTN, hypertension; cHL, classical Hodgkin lymphoma; HyperCVAD, hyperfractionated cyclophosphamide, vincristine, doxorubicin, dexamethasone; RT, radiotherapy; GDP, gemcitabine, dexamethasone, cisplatin; O, obinutuzumab; MINE, mesna, ifosfamide, mitoxantrone, etoposide; R, rituximab.

**Table 2:** ViPOR treatment course, response, and clinical outcomes.

Case	Time to Interim Response (Cycle)	Interim Response Assessment	Total ViPOR Cycles	Last Response Assessment	Subsequent Therapies and Responses	Clinical Outcomes and Complications
1	2	PR	5	CR	Allo-SCT (CR)	RT administered to residual nodal disease after interim assessment; ViPOR continued concurrently. In CR at 13 months post-allo-SCT.
2	Early death during Cycle 1	-	1	-	-	Exitus on cycle 1, day 17 due to lymphoma progression
3	3	PR	4	CR	Allo-SCT (CR)	RT administered to residual nodal disease after interim assessment; ViPOR continued concurrently. In CR at 8 months post–allo-SCT
4	2	CR	2	CR	Allo-SCT (CR)	In CR at 10 months post-allo-SCT
5	2	PD	2	PD	RT (PD), Selinexor (PD)	Exitus due to lymphoma progression
6	3	CR	4	CR	-	Alive, off follow-up due to CNS aspergillosis-related hemiplegia; not eligible for further therapy
7	2	SD	2	SD	-	Exitus due to pulmonary thromboembolism
8	2	PR	3	PD	-	Exitus due to lymphoma progression
9	3	CR	6	CR	Allo-SCT	Alive on day +67 post-allo-SCT
10	2	PR	2	CR	Allo-SCT	RT administered to residual nodal disease after 2 cycles of ViPOR. Exitus on Day +31 post-allo-SCT due to sepsis and septic shock
11	3	SD	3	SD	RT (PD)	Poor performance status and advanced age; received palliative RT and died due to lymphoma progression
12	2	SD	4	SD	-	Refractory to ViPOR; referred to a Phase 1 clinical trial (NCT07308132)
13	3	CR	3	CR	Allo-SCT	Alive on Day +71 post-allo-SCT
14	2	PD	2	PD	-	Alive with progressive disease; no subsequent therapy initiated yet

Abbreviations: ViPOR, venetoclax, ibrutinib, prednisone, obinutuzumab, lenalidomide; PR, partial response; CR, complete response; SD, stable disease; PD, progressive disease; RT, radiotherapy; SCT, stem cell transplantation; allo-SCT, allogeneic stem cell transplantation; CNS, central nervous system.

**Table 3:** Response and clinical outcomes according to cell-of-origin subtype.

Outcome	DLBCL, ABC (n = 8)	DLBCL, GC (n = 4)
CR at end of ViPOR, n (%)	5 (62.5)	0 (0)
SD at end of ViPOR, n (%)	2 (25.0)	1 (25.0)
PD at end of ViPOR, n (%)	1 (12.5)	3 (75.0)
ORR (CR + PR), n (%)	5 (62.5)	0 (0)
Bridged to allo-SCT, n (%)	5 (62.5)	0 (0)
Deaths during follow-up, n (%)	3 (37.5)	4 (100)
Alive at last follow-up, n (%)	5 (62.5)	0 (0)

Abbreviations: ABC, activated B-cell subtype; GC, germinal center subtype; CR, complete response; SD, stable disease; PD, progressive disease; ORR, overall response rate; allo-SCT, allogeneic stem cell transplantation.

**Table 4:** Adverse events and prophylaxis during ViPOR.

Case	Adverse Events (Grade/Type)	TLS (Tumor Lysis Syndrome)	Prophylaxis Given
1	Neutropenia, hypokalemia, diarrhea—all grade 2	No	Valacyclovir, TMP-SMX, entecavir
2	None	No	Valacyclovir, TMP-SMX
3	Neutropenia grade 3, Hypokalemia, diarrhea—both grade 2	No	Valacyclovir, TMP-SMX
4	Neutropenia grade 3 Diarrhea grade 2	No	Valacyclovir, TMP-SMX
5	Neutropenia grade 3	No	Valacyclovir, TMP-SMX
6	CNS aspergillosis Neutropenia grade 2	No	Valacyclovir, TMP-SMX
7	Pulmonary embolism	No	Valacyclovir, TMP-SMX, Enoxaparin
8	Thrombocytopenia, neutropenia—both grade 2 Soft tissue infection grade 2	No	Acyclovir, TMP-SMX
9	Neutropenia grade 2, diarrhea grade 2	No	Valacyclovir, TMP-SMX, entecavir, fluconazole
10	None	No	Valacyclovir, TMP-SMX
11	None	No	Valacyclovir, TMP-SMX
12	Neutropenia, thrombocytopenia, diarrhea, hypokalemia—all grade 3	No	Valacyclovir, TMP-SMX
13	None	No	Valacyclovir, TMP-SMX
14	None	No	Valacyclovir, TMP-SMX

Abbreviations: ViPOR, venetoclax, ibrutinib, prednisone, obinutuzumab, lenalidomide; TLS, tumor lysis syndrome; CNS, central nervous system; TMP-SMX, trimethoprim–sulfamethoxazole.

## Data Availability

The data supporting the findings of this study are available from the corresponding author upon reasonable request.

## References

[1] Silkenstedt E., Salles G., Campo E., Dreyling M. (2024). B-cell non-Hodgkin lymphomas. Lancet.

[2] Crump M., Neelapu S.S., Farooq U., Van Den Neste E., Kuruvilla J., Westin J., Link B.K., Hay A., Cerhan J.R., Zhu L. (2017). Outcomes in refractory diffuse large B-cell lymphoma: Results from the international SCHOLAR-1 study. Blood.

[3] Bandy S., Wu A., Gratie D., Horblyuk R., Repetny K., Fanale M., Mitchell B., Liu N. (2024). A Systematic Literature Review of Clinical Outcomes in Patients with Relapsed or Refractory Diffuse Large B-Cell Lymphoma Treated in the Third-Line or Later Setting. Blood.

[4] Tun A.M., Wang Y., Maliske S., Micallef I., Inwards D.J., Habermann T.M., Porrata L., Paludo J., Bisneto J.V., Rosenthal A. (2024). Autologous stem cell transplant in fit patients with refractory or early relapsed diffuse large B-cell lymphoma that responded to salvage chemotherapy. Haematologica.

[5] Di Blasi R., Le Gouill S., Bachy E., Cartron G., Beauvais D., Le Bras F., Gros F.-X., Choquet S., Bories P., Feugier P. (2022). Outcomes of patients with aggressive B-cell lymphoma after failure of anti-CD19 CAR T-cell therapy: A DESCAR-T analysis. Blood.

[6] Profitós-Pelejà N., Santos J.C., Marín-Niebla A., Roué G., Ribeiro M.L. (2022). Regulation of B-Cell Receptor Signaling and Its Therapeutic Relevance in Aggressive B-Cell Lymphomas. Cancers.

[7] Ondrisova L., Mraz M. (2020). Genetic and Non-Genetic Mechanisms of Resistance to BCR Signaling Inhibitors in B Cell Malignancies. Front. Oncol..

[8] Melani C., Lakhotia R., Pittaluga S., Phelan J.D., Huang D.W., Wright G., Simard J., Muppidi J., Thomas C.J., Ceribelli M. (2024). Combination Targeted Therapy in Relapsed Diffuse Large B-Cell Lymphoma. N. Engl. J. Med..

[9] Oellerich T., Wurm-Kuczera R., Sahay S., Wang M., Ali S., Serin N., Kouidri K., Tahiri D., Scheich S., Brunnberg U. (2025). Efficacy and safety of vipor (P) in relapsed or refractory large B-cell lymphoma. Blood.

[10] Wurm-Kuczera R., Kouidri K., Wang M., Ali S., Sahay S., Winkler D., Leskien A.-K., Pudasaini S., Kauer J., Krämer I. (2024). Real-World Evidence of Venetoclax, Ibrutinib, Prednisone, Obinutuzumab, Lenalidomide with or without Polatuzumab (VIPOR(P)) for Relapsed/Refractory Large B-Cell Lymphoma. Blood.

[11] Dalela D., Lakhotia R., Melani C., Pittaluga S., Phelan J.D., Muppidi J.R., Evans S., Pradhan A., Tadese A., Morrison C. (2024). A Pilot Study of Venetoclax, Ibrutinib, Prednisone, Obinutuzumab, and Lenalidomide (VIPOR) for Diffuse Large B-Cell Lymphoma Involving the Central Nervous System. Blood.

[12] Hans C.P. (2004). Confirmation of the molecular classification of diffuse large B-cell lymphoma by immunohistochemistry using a tissue microarray. Blood.

[13] Wilson W.H., Gerecitano J.F., Goy A., de Vos S., Kenkre V.P., Barr P.M., Blum K.A., Shustov A.R., Advani R.H., Lih J. (2012). The Bruton’s Tyrosine Kinase (BTK) Inhibitor, Ibrutinib (PCI-32765), Has Preferential Activity in the ABC Subtype of Relapsed/Refractory De Novo Diffuse Large B-Cell Lymphoma (DLBCL): Interim Results of a Multicenter, Open-Label, Phase 2 Study. Blood.

[14] Wilson W.H., Young R.M., Schmitz R., Yang Y., Pittaluga S., Wright G., Lih C.-J., Williams P.M., Shaffer A.L., Gerecitano J. (2015). Targeting B cell receptor signaling with ibrutinib in diffuse large B cell lymphoma. Nat. Med..

[15] Balasubramanian S., Crowley R., Sirisawad M., Thiemann P., Chen J., Buggy J.J. (2011). The Bruton’s Tyrosine Kinase (BTK) Inhibitor PCI-32765 Inhibits Growth of ABC DLBCL Tumors In Vivo and in Vitro by Preventing Activation of Pro-Survival NF-κB pathways. Blood.

[16] Bertram K., Leary P.J., Boudesco C., Fullin J., Stirm K., Dalal V., Zenz T., Tzankov A., Müller A. (2022). Inhibitors of Bcl-2 and Bruton’s tyrosine kinase synergize to abrogate diffuse large B-cell lymphoma growth in vitro and in orthotopic xenotransplantation models. Leukemia.

[17] Melchor J., Garcia-Lacarte M., Grijalba S.C., Arnaiz-Leché A., Pascual M., Panizo C., Blanco O., Segura V., Novo F.J., Valero J.G. (2023). Venetoclax improves CD20 immunotherapy in a mouse model of MYC/BCL2 double-expressor diffuse large B-cell lymphoma. J. Immunother. Cancer.

[18] Yang Y., Shaffer A.L., Emre N.C.T., Ceribelli M., Zhang M., Wright G., Xiao W., Powell J., Platig J., Kohlhammer H. (2012). Exploiting Synthetic Lethality for the Therapy of ABC Diffuse Large B Cell Lymphoma. Cancer Cell.

[19] Zhang L., Kosek J., Wang M., Heise C., Schafer P.H., Chopra R. (2013). Lenalidomide efficacy in activated B-cell-like subtype diffuse large B-cell lymphoma is dependent upon IRF 4 and cereblon expression. Br. J. Haematol..

[20] Choi J., Ceribelli M., Phelan J.D., Häupl B., Huang D.W., Wright G., Hsiao T., Morris V.M., Ciccarese F., Wang B. (2023). Molecular Mechanism of Action of Glucocorticoids in Lymphoma Therapy. Blood.

[21] Edelmann J., Dokal A., Holzmann K., Britton D.J., Vilventhraraja E., Smith R.J., Cragg M.S., Braun A., Döhner H., Cutillas P. (2019). Rituximab and Obinutuzumab Induce Direct B-Cell Death Via B-Cell Receptor (BCR) Signaling, but Rituximab Elicits Stronger BCR-Derived Pro-Survival Signals Diminishing Apoptosis. Blood.

[22] Melani C., Lakhotia R., Pittaluga S., Miljkovic M.D., Muppidi J.R., Portell C.A., Farah R., Lee S.T., Juanitez A.M., Chou L.L. (2019). Phase 1b Study of Vipor (Venetoclax, Ibrutinib, Prednisone, Obinutuzumab, and Lenalidomide) in Relapsed/Refractory B-Cell Lymphoma: Safety, Efficacy and Molecular Analysis. Blood.

[23] Melani C., Lakhotia R., Pittaluga S., Miljkovic M.D., Phelan J.D., Muppidi J.R., Thomas C.J., Ceribelli M., Tosto F.A., Portell C.A. (2020). Phase 1b/2 Study of Vipor (Venetoclax, Ibrutinib, Prednisone, Obinutuzumab, and Lenalidomide) in Relapsed/Refractory B-Cell Lymphoma: Safety, Efficacy and Molecular Analysis. Blood.

[24] Melani C., Lakhotia R., Pittaluga S., Phelan J.D., Simard J., Muppidi J.R., Thomas C.J., Ceribelli M., Tosto F.A., Farah R.J. (2022). Venetoclax, Ibrutinib, Prednisone, Obinutuzumab, and Lenalidomide (ViPOR) in Relapsed and Refractory Follicular Lymphoma: Analysis of Safety, Efficacy, and Minimal Residual Disease. Blood.

[25] Melani C., Lakhotia R., Pittaluga S., Phelan J.D., Huang D.W., Wright G., Simard J., Muppidi J.R., Thomas C.J., Ceribelli M. (2023). Phase Ib/II Study of Multi-Targeted Therapy with Venetoclax, Ibrutinib, Prednisone, Obinutuzumab, and Lenalidomide (ViPOR) in Relapsed/Refractory (R/R) Diffuse Large B-Cell Lymphoma (DLBCL). Blood.

[26] Melani C.J., Lakhotia R., Pittaluga S., Phelan J.D., Yang Y., Davies-Hill T., Simard J., Muppidi J., Huang D.W., Thomas C.J. (2023). Venetoclax, ibrutinib, prednisone, obinutuzumab, and lenalidomide (ViPOR) in relapsed/refractory (R/R) and treatment-naïve (TN) mantle cell lymphoma (MCL). Hematol. Oncol..

[27] Melani C., Lakhotia R., Pittaluga S., Phelan J.D., Muppidi J., Gordon M., Yang Y., Xu W., Davies-Hill T., Huang D.W. (2025). 316|MULTI-TARGETED THERAPY WITH VIPOR-P IN RELAPSED/REFRACTORY DIFFUSE LARGE B-CELL LYMPHOMA: UPDATED ANALYSIS OF EFFICACY AND MINIMAL RESIDUAL DISEASE. Hematol. Oncol..

